# Clinical utility of the pancreatitis activity scoring system in severe acute pancreatitis

**DOI:** 10.3389/fphys.2022.935329

**Published:** 2022-08-22

**Authors:** Zetao Yu, Qingqiang Ni, Peng Zhang, Hongtao Jia, Faji Yang, Hengjun Gao, Huaqiang Zhu, Fangfeng Liu, Xu Zhou, Hong Chang, Jun Lu

**Affiliations:** ^1^ Department of Hepatobiliary Surgery, Shandong Provincial Hospital Affiliated to Shandong First Medical University, Department of Hepatobiliary Surgery, Shandong Provincial Hospital, Shandong University, Shandong, China; ^2^ ICU, Shandong Provincial Hospital Affiliated to Shandong First Medical University, ICU, Shandong Provincial Hospital, Shandong University, Shandong, China

**Keywords:** severe acute pancreatitis, pancreatitis activity scoring system, persistent organ failure, poor prognosis, in-hospital mortality

## Abstract

**Objective:** To analyze clinical utility of pancreatitis activity scoring system (PASS) in prediction of persistent organ failure, poor prognosis, and in-hospital mortality in patients with moderately severe acute pancreatitis (MSAP) or severe acute pancreatitis (SAP) admitted to the intensive care unit (ICU).

**Methods:** The study included a total of 140 patients with MSAP and SAP admitted to the ICU of Shandong Provincial Hospital from 2015 to 2021. The general information, biochemical indexes and PASS scores of patients at ICU admission time were collected. Independent risk factors of persistent organ failure, poor prognosis and in-hospital mortality were analyzed by binary logistic regression. Through receiver operating characteristic curve (ROC), the predictive ability of lactic acid, procalcitonin, urea nitrogen, PASS, and PASS in combination with urea nitrogen for the three outcomes was compared. The best cut-off value was determined.

**Results:** Binary logistic regression showed that PASS might be an independent risk factor for patients with persistent organ failure (odds ratio [OR]: 1.027, 95% confidence interval [CI]: 1.014–1.039), poor prognosis (OR: 1.008, 95% CI: 1.001–1.014), and in-hospital mortality (OR: 1.009, 95% CI: 1.000–1.019). PASS also had a good predictive ability for persistent organ failure (area under the curve (AUC) = 0.839, 95% CI: 0.769–0.910) and in-hospital mortality (AUC = 0.780, 95% CI: 0.669–0.891), which was significantly superior to lactic acid, procalcitonin, urea nitrogen and Ranson score. PASS (AUC = 0.756, 95% CI: 0.675–0.837) was second only to urea nitrogen (AUC = 0.768, 95% CI: 0.686–0.850) in the prediction of poor prognosis. Furthermore, the predictive power of urea nitrogen in combination with PASS was better than that of each factor for persistent organ failure (AUC = 0.849, 95% CI: 0.779–0.920), poor prognosis (AUC = 0.801, 95% CI: 0.726–0.876), and in-hospital mortality (AUC = 0.796, 95% CI: 0.697–0.894).

**Conclusion:** PASS was closely correlated with the prognosis of patients with MSAP and SAP. This scoring system may be used as a common clinical index to measure the activity of acute pancreatitis and evaluate disease prognosis.

## Introduction

Acute pancreatitis (AP) is a disease with localized pancreatic or systemic inflammatory response primarily due to abnormal activation of pancreatin. Biliary and alcoholic AP are relatively common. In developed countries, AP incidence can reach 34/100,000 people, which is progressively increasing each year (Leppäniemi et al., 2019; Boxhoorn et al., 2020). According to the revised Atlanta classification, this disease can be divided into mild acute pancreatitis (MAP), moderately severe acute pancreatitis (MSAP) and severe acute pancreatitis (SAP) (Banks et al., 2013; Colvin et al., 2020). MAP has mild self-limiting symptoms and a short clinical duration. While this type of AP can heal itself after supportive treatment of its symptoms, 20% of MAP cases may have organ failure and subsequently progress to MSAP and SAP with a mortality rate of 20–40%. Therefore, early prediction and intervention of AP may reduce organ failure occurrence and improve disease prognosis (Garg and Singh, 2019).

**FIGURE 1:**
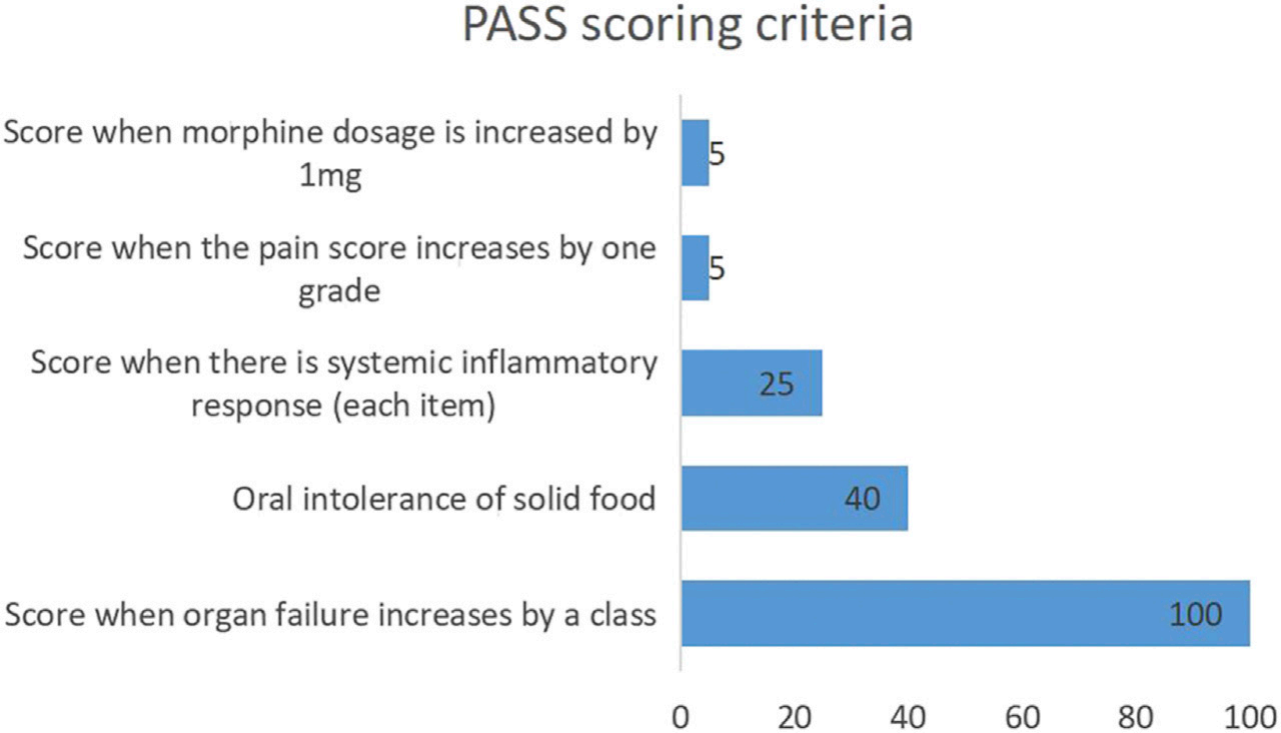
PASS scoring criteria. PASS: pancreatitis activity scoring system.

Studies have found that indicators such as urea nitrogen, high density lipoprotein cholesterol, and procalcitonin are closely correlated with AP severity (Wu et al., 2011; Zhang et al., 2017; Vitale et al., 2019; Li et al., 2021; Pando et al., 2021). Additionally, urea nitrogen has been included in Ranson score to evaluate the AP progression and prognosis. In order to better evaluate and predict AP, a new pancreatitis activity scoring system (PASS) was proposed by international experts in 2017 (Wu et al., 2017). There are five scoring items, including organ failure (100 points for organ failure in each system), oral intolerance to solid food (40 points), systemic inflammatory response (25 points for each item), abdominal pain (5 points for each grade increase), and opioid application (5 points for each mg) (Figure 1). Some studies have also investigated the relationships of PASS with readmission rate of AP, infectious pancreatic necrosis, and other complications (Buxbaum et al., 2018; Ke et al., 2018; Lew et al., 2018; Boxhoorn et al., 2020; Paragomi et al., 2021; Thiruvengadam et al., 2021). However, there are few studies on the relationships of PASS with persistent organ failure (POF), poor prognosis and in-hospital mortality. In this study, a case-control study was employed to analyze the relationships of the initial PASS with POF, poor prognosis and in-hospital mortality in MSAP and SAP patients admitted to the ICU for the evaluation of its clinical utility.

## Materials and methods

### Data collection

This study enrolled 140 patients with MSAP and SAP who were admitted to the intensive care unit of Shandong Provincial Hospital from 2015 to 2021. Inclusion criteria were as follows: 1) age >16 years; 2) presence of AP diagnostic criteria; 3) presence of MSAP and SAP diagnostic criteria in the revised Atlanta classification; 4) presence of all information related to PASS score.

Patients with MAP are not associated with organ failure and local or systemic complications, and usually recovers within 1–2 weeks with very low mortality. All MAP patients were not admitted into ICU, which were excluded. The guidelines of ICU admission were consensus on which patients should be referred to intensive care and were assessed by a professional ICU attending physician. ICU inclusion criteria: Firstly, clinical manifestations and signs as well as biochemical changes of AP, associated with either organ failure, local or systemic complications; Secondly, patients need intensive care, keep monitoring and evaluating the vital signs. Exclusion criteria for ICU: organ failure induced by non-AP.

These clinical data were collected from our hospital’s electronic case system. This study was approved by the Ethics Committee of Shandong Provincial Hospital, and met the requirements in the Declaration of Helsinki. Written informed consent forms were obtained from all patients.

The demographic information of patients, including age, gender, heart rate, body temperature, systolic blood pressure, smoking history, drinking history, and diabetes history was collected. Hematological examination results of patients admitted to the ICU, including red blood cells, white blood cells, neutrophils, mean platelet volume, glutamic oxaloacetic transaminase, albumin, urea nitrogen, creatinine, activated partial prothrombin time, prothrombin time, triglyceride, total cholesterol, procalcitonin, lactic acid, and blood amylase, the first PASS and Ranson scores were also included.

### Diagnostic criteria

Diagnosis of acute pancreatitis could be made when two of the following three items were present: 1) abdominal pain associated with pancreatitis; 2) increase of blood amylase or blood lipase to a level higher than 3 times the normal value; 3) imaging manifestations of pancreatitis (Boxhoorn et al., 2020). MARSHALL score was used as the diagnostic criteria for organ failure. Any system scores greater than or equal to two could be regarded as organ failure. POF was confirmed when the duration was more than 48 h. Transient organ failure was considered when the duration was less than 48 h (Abu Omar et al., 2019). The PASS score was assessed within 24 h after admission to ICU by specialized attending physicians who admitted the patient into ICU. Each patient underwent CT/MRI scan. A radiologist may also participant in the evaluation of CT/MRI scans, but the final CT/MRI evaluation was completed by the ICU specialist attending physician after a comprehensive analysis. Patients could be divided into the mortality group and survival group according to the presence of in-hospital mortality. Poor prognosis was defined as the need for transference to a higher-level care hospital due to poor improvement or death during hospitalization. Patients could be divided into the recovery group and non-recovery group according to the presence of poor prognosis.

### Statistical analysis

In this study, variables that conformed to normal distribution were expressed as mean ± standard deviation (SD). Variables that did not conform to normal distribution were expressed as median and interquartile range (IQR). Categorical variables were expressed as number of cases and percentages. In univariate analysis, Student’s t-test, Mann-Whitney U test and Fisher’s exact test were used respectively. *p* < 0.05 was considered statistically significant. The variables with *p* < 0.05 in univariate analysis were taken as covariates and included in the multivariate analysis. Through binary logistic regression, independent risk factors for POF, in-hospital mortality and poor prognosis of patients with MSAP and SAP were analyzed. The evaluation efficiency, sensitivity, specificity, and best cut-off value of procalcitonin, urea nitrogen, lactic acid, PASS, and PASS in combination with urea nitrogen for POF, mortality and overall prognosis of patients with MSAP and SAP were compared by the receiver operating characteristic (ROC) curve.

## Results

### Demographic information analysis

A total of 140 patients were enrolled in this study, including 85 males (60.7%), with a median age of 39.5 (IQR: 33.3–58.0) years, and 70 were from the emergency department, 18 were from the Department of General Gastroenterology, and 52 were transferred from other hospitals. The mean time from onset to ICU admission around these patients was 3.0 (2.0,5.8) days. There were 86 patients with POF, including 60 males (69.8%), with a median age of 44.5 (IQR: 35.8–64.5) years. A total of 12 patients died, including nine males (75%), with a median age of 53.0 (IQR: 37.0–71.5) years. There were 46 patients with poor prognosis, including 29 males (61.7%), and the median age was 50.0 (IQR: 35.0–67.0) years. In the transient organ failure and POF groups, except for gender (25 [46.3%] vs 60 [69.8%]), age (35.5 [28.8–41.8] vs 44.5 [35.8–64.5]), and systolic blood pressure (126.6 ± 18.4 vs 134.6 ± 24.2), the rest of the general information showed no statistically significant difference. There was no statistically significant difference in demographic information between the mortality group and the non-mortality group. In the recovery group and the non-recovery group, except for age (37.0 [21.5–50.5] vs 50.0 [35.0–67.0]), all the other demographic information also showed no statistically significant difference (Table 1).

**TABLE 1:** Demographic information of AP patients.

Variable	Total population	POF group	Mortality group	Non-recovery group
Total number of people	140	86	12	46
Gender (male)	85 (60.7%)	60 (69.8%)*	9 (75%)	29 (61.7%)
Fever	57 (40.7%)	40 (46.5%)	8 (66.7%)	18 (38.3%)
Smoking	60 (42.9%)	40 (46.5%)	4 (33.3%)	19 (40.4%)
Alcohol drinking	63 (45.0%)	42 (48.8%)	4 (33.3%)	21 (44.7%)
Diabetes	19 (13.6%)	11 (12.8%)	0 (0%)	3 (6.4%)
Age	39.5 (33.3–58.5)	44.5 (35.8–64.5)*	53.0 (37.0–71.5)	50.0 (35.0–67.0)*
Heart rate	111.7 ± 19.6	113.9 ± 19.7	113.3 ± 20.2	113.9 ± 18.7
Systolic blood pressure	131.5 ± 22.4	134.6 ± 24.2*	130.6 ± 32.9	131.7 ± 25.3

*There was a statistically significant difference between the two groups (*p* < 0.05).

### Correlation between PASS and POF

Univariate analysis of the hematological indexes and PASS of the transient organ failure and POF groups showed that the mean platelet volume, glutamic oxaloacetic transaminase, urea nitrogen, creatinine, prothrombin time, procalcitonin and PASS were relatively high, and triglycerides and total cholesterol were relatively low. There was no significant difference in other indicators (Table 2).

**TABLE 2:** Univariate analysis of the POF group and TOF group.

Variable	TOF group	POF group	*p* Value
MPV	10.6 ± 1.1	11.2 ± 1.4	0.003
AST	24.5 (17.0–41.3)	50.5 (28.0–105.3)	<0.001
BUN	4.9 (3.5–7.1)	10.1 (6.1–15.4)	<0.001
CREA	60.0 (43.2–80.2)	95.9 (58.3–246.7)	<0.001
PT	14.6 (13.7–15.7)	15.6 (14.6–17.3)	0.003
TG	7.1 (3.0–10.9)	2.8 (1.5–7.1)	<0.001
TC	6.4 (4.8–10.1)	3.9 (2.7–5.8)	<0.001
PCT	0.7 (0.3–1.9)	2.9 (1.1–13.8)	<0.001
PASS score	127.5 (95.0–215.0)	227.5 (208.8–300.0)	<0.001

Only the variables with statistically significant differences were shown (*p* < 0.05). AST, glutamic oxaloacetic transaminase; BUN, urea nitrogen; CREA, creatinine; MPV, mean platelet volume; PCT, procalcitonin; PT, prothrombin time; TC, total cholesterol; TG, triglyceride.

Using the variables with statistically significant differences in the univariate analysis as the arguments and POF as the dependent variable, binary logistic regression analysis showed that age (OR: 1.060, 95% CI: 1.011–1.111), gender (OR: 0.190, 95% CI: 0.049–0.735), mean platelet volume (OR: 1.851, 95% CI: 1.133–3.026) and PASS (OR: 1.027, 95% CI: 1.014–1.039) were independently correlated with POF. These differences were statistically significant (*p* < 0.05, Table 6).

**FIGURE 2:**
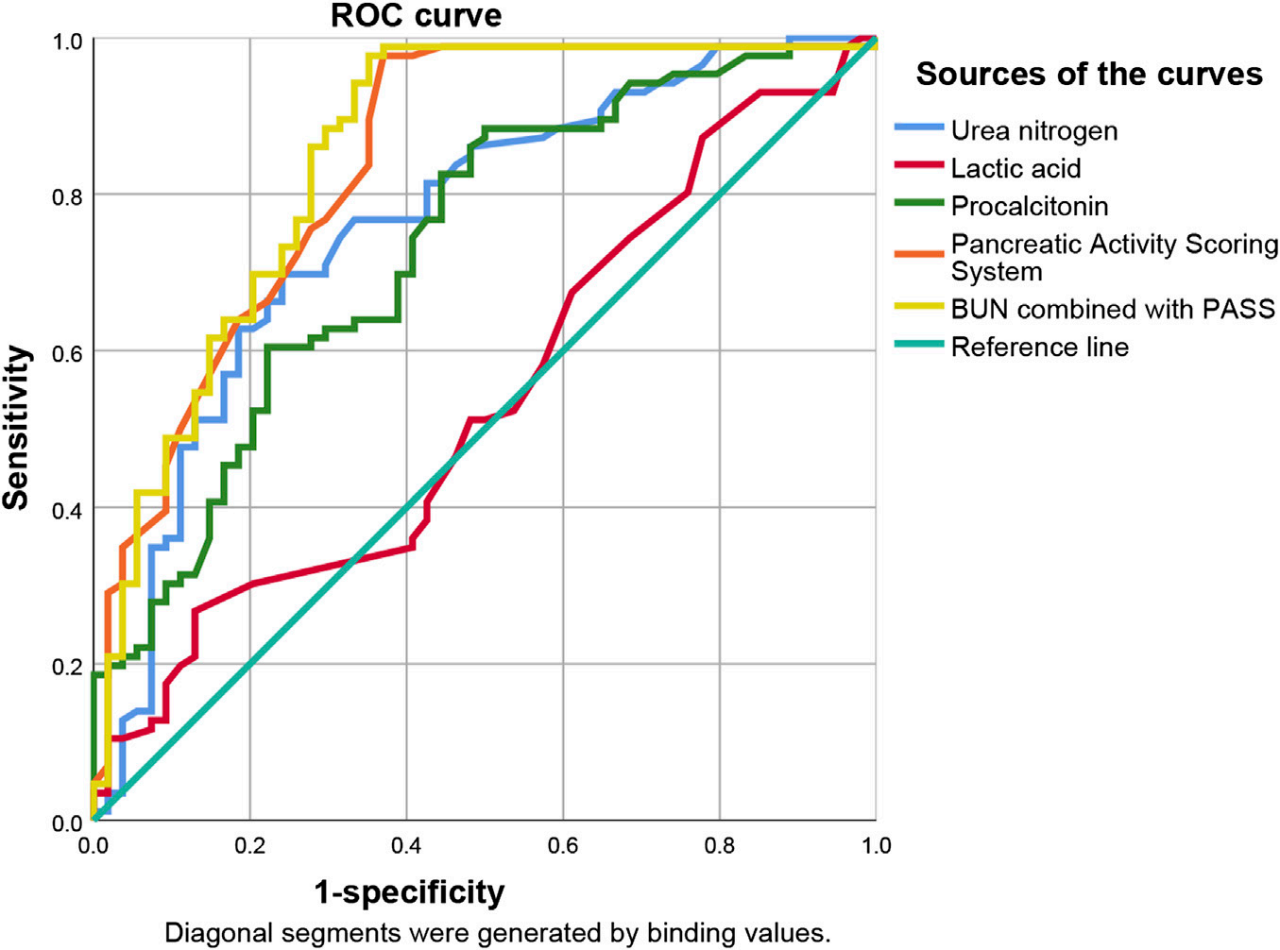
Predictive efficacy for POF of clinical parameters of AP. POF: persistent organ failure; AP: acute pancreatitis.

**FIGURE 3:**
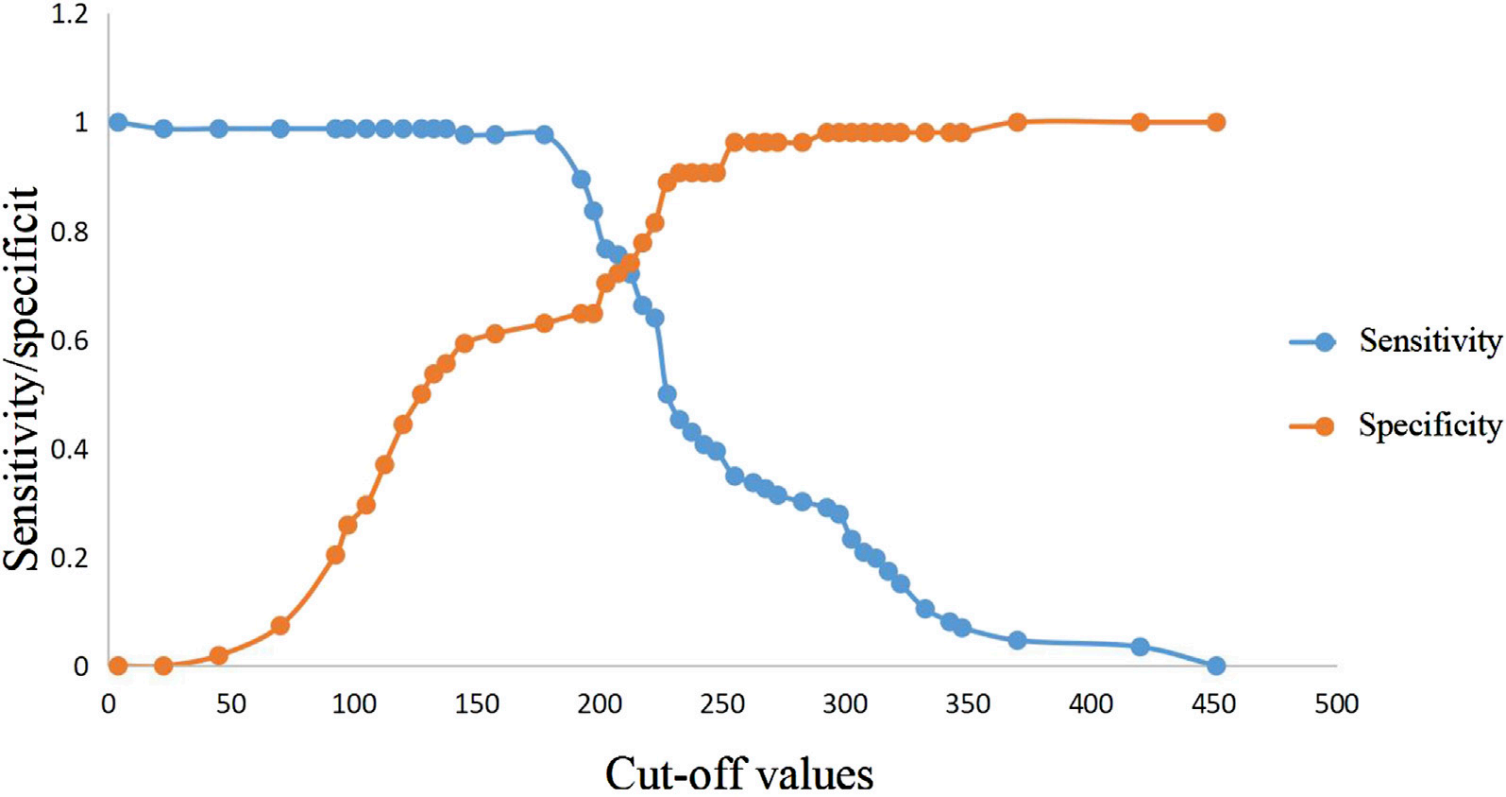
Sensitivity and specificity curves of PASS score in POF prediction in the presence of different cut-off values. PASS: pancreatitis activity scoring system; POF: persistent organ failure.

ROC curve analysis showed that lactic acid (AUC = 0.541, 95% CI: 0.443–0.639), procalcitonin (AUC = 0.734, 95% CI: 0.649–0.819), urea nitrogen (AUC = 0.764, 95% CI: 0.681–0.847), PASS (AUC = 0.839, 95% CI: 0.769–0.910), and urea nitrogen combined with PASS (AUC = 0.849, 95% CI: 0.779–0.920) are predictors of POF. The sensitivity of lactic acid, procalcitonin, urea nitrogen and PASS in predicting POF were 0.267, 0.884, 0.698 and 0.977, respectively while their specificity were 0.870, 0.500, 0.759 and 0.630, respectively. The best cut-off values were 2.3 mmol/L, 0.7 ng/ml, 7.1mmol, and 177.5 points, respectively (Table 4; Figures 2, 3).

### Correlation between PASS and poor prognosis

Comparing the hematological indexes of the non-recovery group and the recovery group with PASS, the mean platelet volume, glutamic oxaloacetic transaminase, blood urea nitrogen, creatinine, prothrombin time, procalcitonin, lactic acid and PASS were relatively high. In contrast, total cholesterol was relatively low. There was no significant difference in other indicators (Table 3).

**TABLE 3:** Univariate analysis of recovery group and non-recovery group.

Variable	Recovery group	Non-recovery group	*p* Value
MPV	10.7 ± 1.2	11.4 ± 1.4	0.006
AST	28.0 (20.0–48.0)	63.0 (31.0–115.0)	<0.001
BUN	6.1 (4.2–9.3)	13.4 (7.1–18.0)	<0.001
CREA	62.0 (50.4–92.9)	149.3 (63.2–273.0)	<0.001
PT	14.8 (14.0–16.0)	15.8 (14.6–17.4)	0.009
TC	5.8 (3.6–7.8)	3.3 (2.3–5.7)	<0.001
PCT	1.1 (0.4–3.7)	4.1 (1.8–20.5)	<0.001
Lac	1.4 (1.0–2.1)	2.0 (1.3–3.2)	0.001
PASS score	200.0 (115.0–225.0)	250.0 (215.0–315.0)	<0.001

Only the variables with statistically significant differences were shown (*p* < 0.05). AST, glutamic oxaloacetic transaminase; BUN, urea nitrogen; CREA, creatinine; Lac, lactic acid; MPV, mean platelet volume; PCT, procalcitonin; PT, prothrombin time; TC, total cholesterol.

Using variables with statistically significant differences in univariate analysis as the arguments and the patients’ prognosis as the dependent variable, binary logistic regression analysis showed that lactic acid (OR: 1.611, 95% CI: 1.090–2.380) and PASS (OR: 1.008, 95% CI: 1.001–1.014) were independently correlated with poor prognosis (*p* < 0.05, Table 6).

**FIGURE 4:**
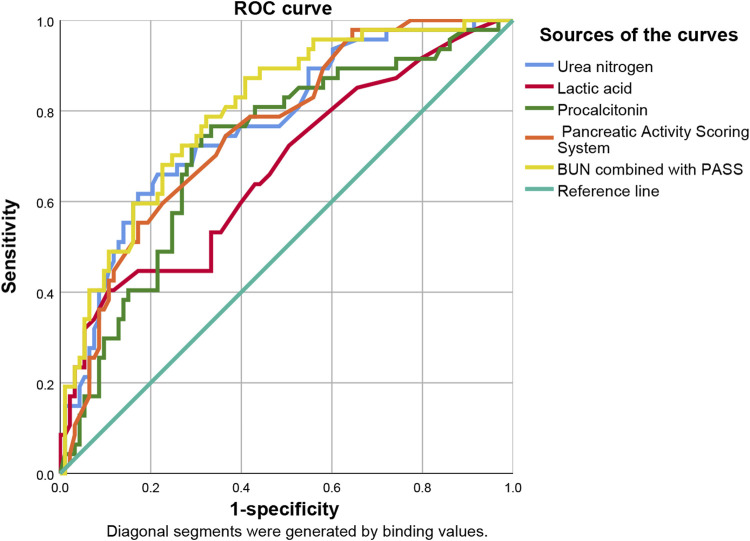
Predictive efficacy for poor prognosis of clinical parameters of AP. AP: acute pancreatitis.

**FIGURE 5:**
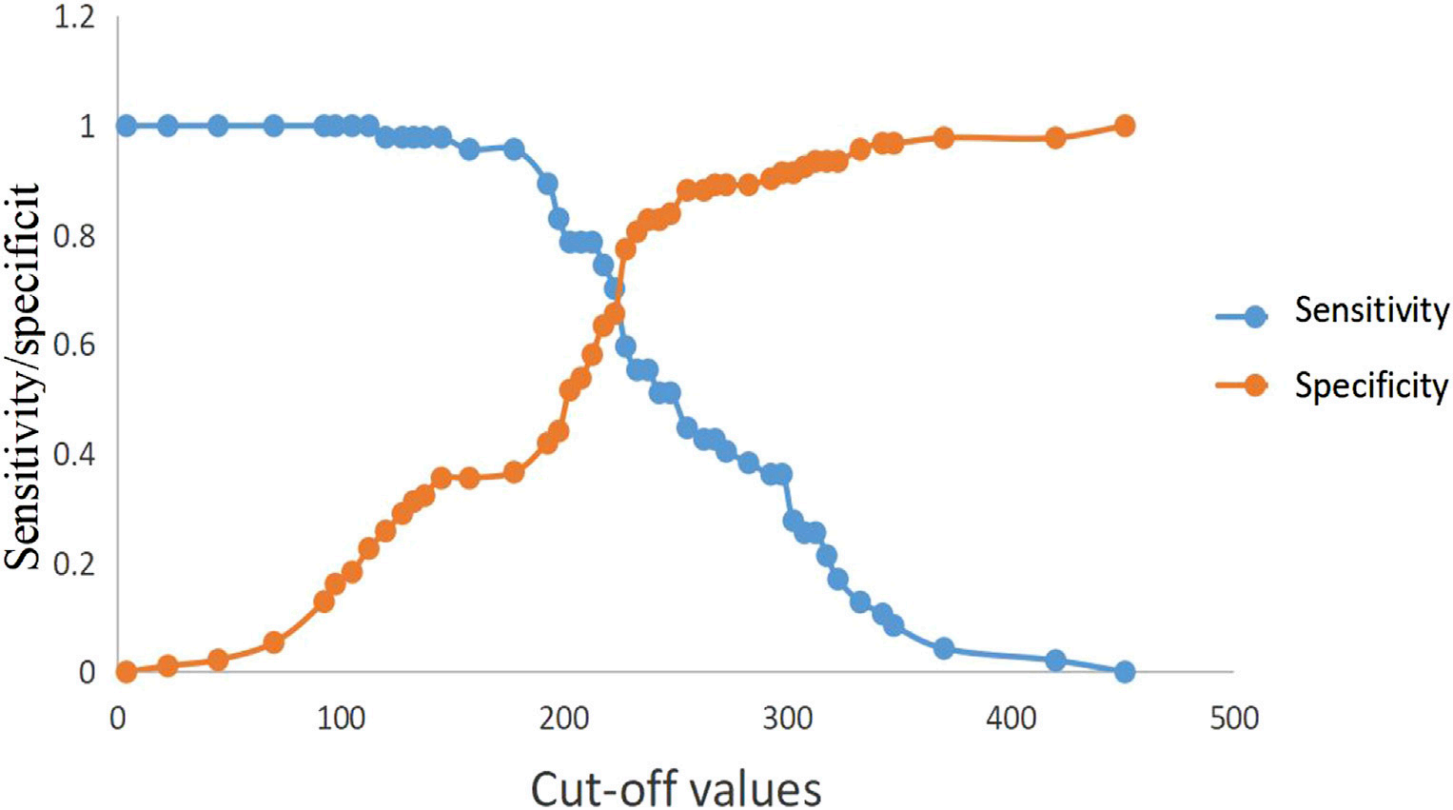
Sensitivity and specificity curves of PASS scores in poor prognosis prediction in the presence of different cut-off values. PASS: pancreatitis activity scoring system.

ROC curve analysis showed that lactic acid (AUC = 0.672, 95% CI: 0.575–0.768), procalcitonin (AUC = 0.718, 95% CI: 0.628–0.808), urea nitrogen (AUC = 0.768, 95% CI: 0.686–0.850), PASS (AUC = 0.756, 95% CI: 0.675–0.837) and urea nitrogen combined with PASS (AUC = 0.801, 95% CI: 0.726–0.876) had predictive values for poor prognosis. The sensitivity of lactic acid, procalcitonin, urea nitrogen and PASS in poor prognosis prediction were 0.404, 0.745, 0.617 and 0.553, respectively while their specificity were 0.892, 0.688, 0.828 and 0.828, respectively. The best cut-off values were 2.4 mmol/L, 2.0 ng/ml, 11.4 mmol/L and 237.5 points, respectively (Table 4; Figures 4, 5).

**TABLE 4:** ROC curve analysis of potential clinical predictors of AP outcomes.

Variable	AUC	Cut-off level	Sensitivity	Specificity	Youden’s index
Correlation analysis of POF					
LAC	0.541	2.3	0.267	0.870	0.137
PCT	0.734	0.7	0.884	0.500	0.384
BUN	0.764	7.1	0.698	0.759	0.457
PASS	0.839	177.5	0.977	0.630	0.607
BUN combined with PASS	0.849	——	0.977	0.648	0.625
Correlation analysis of poor prognosis					
LAC	0.672	2.4	0.404	0.892	0.296
PCT	0.718	2.0	0.745	0.688	0.433
BUN	0.768	11.4	0.617	0.828	0.445
PASS	0.756	237.5	0.553	0.828	0.381
BUN combined with PASS	0.801	——	0.787	0.677	0.464
Correlation analysis of mortality					
LAC	0.729	2.4	0.667	0.836	0.503
PCT	0.710	2.0	0.917	0.586	0.503
BUN	0.774	10.1	0.917	0.687	0.604
PASS	0.780	267.5	0.667	0.828	0.495
BUN combined with PASS	0.796	——	1.000	0.500	0.500

BUN, urea nitrogen; LAC, lactic acid; PCT, procalcitonin.

### Correlation between PASS and in-hospital mortality

Comparing the blood biochemical indexes and PASS between the mortality group and the survival group, the mortality group had relatively higher glutamic oxaloacetic transaminase, urea nitrogen, prothrombin time, procalcitonin, lactic acid and PASS, and relatively low triglyceride and total cholesterol. There was no significant difference in other indicators (Table 5).

**TABLE 5:** Univariate analysis of the mortality group and survival group.

Variable	Survival group	Mortality group	*p* Value
AST	32.0 (20.0–63.0)	80.5 (42.5–186.0)	0.008
BUN	6.9 (4.8–12.3)	14.4 (10.8–19.3)	0.002
PT	15.1 (14.1–16.3)	17.2 (15.5–19.2)	0.011
TG	4.8 (2.3–8.5)	1.3 (0.6–2.1)	<0.001
TC	5.6 (3.2–7.4)	2.7 (1.5–3.2)	<0.001
PCT	1.5 (0.6–9.9)	4.5 (2.6–23.4)	0.016
Lac	1.5 (1.1–2.1)	2.6 (1.5–4.5)	0.009
PASS score	215.0 (140.0–243.8)	287.5 (225.0–318.8)	0.001

Only the variables with statistically significant differences were shown (*p* < 0.05). AST, glutamic oxaloacetic transaminase; BUN, urea nitrogen; Lac, lactic acid; PCT, procalcitonin; PT, prothrombin time; TC, total cholesterol; TG, triglyceride.

In the univariate analysis, the variables with statistically significant differences were taken as the arguments while in-hospital mortality was taken as the dependent variable. Binary logistic regression analysis showed that no independent risk factors were identified. The difference in PASS (OR: 1.009, 95% CI: 1.000–1.019) was not statistically significant (*p* = 0.053, Table 6).

**TABLE 6:** Multivariate analysis of potential clinical predictors of AP outcomes.

Variable	OR (95%CI)	*p* Value
TOF group and POF group		
PASS score	1.027 (1.014–1.039)	<0.001
Age	1.060 (1.011–1.111)	0.016
Gender	0.190 (0.049,0.735)	0.016
Mean platelet volume	1.851 (1.133,3.026)	0.014
Recovery group and non-recovery group		
PASS score	1.008 (1.001–1.014)	0.017
LAC	1.611 (1.090–2.380)	0.017
Mortality group and survival group		
PASS score	1.009 (1.000–1.019)	0.053

Except for the PASS score, only the variables with statistically significant differences were shown (*p* < 0.05). The variables with statistically significant differences in univariate analysis were used as covariates, which were adjusted by binary logistic regression.

LAC, lactic acid.

**FIGURE 6:**
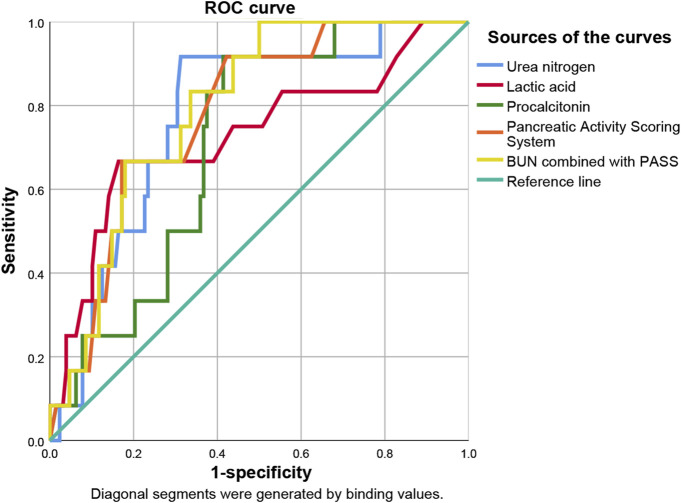
Predictive efficacy for in-hospital mortality of clinical parameters of AP. AP: acute pancreatitis.

**FIGURE 7:**
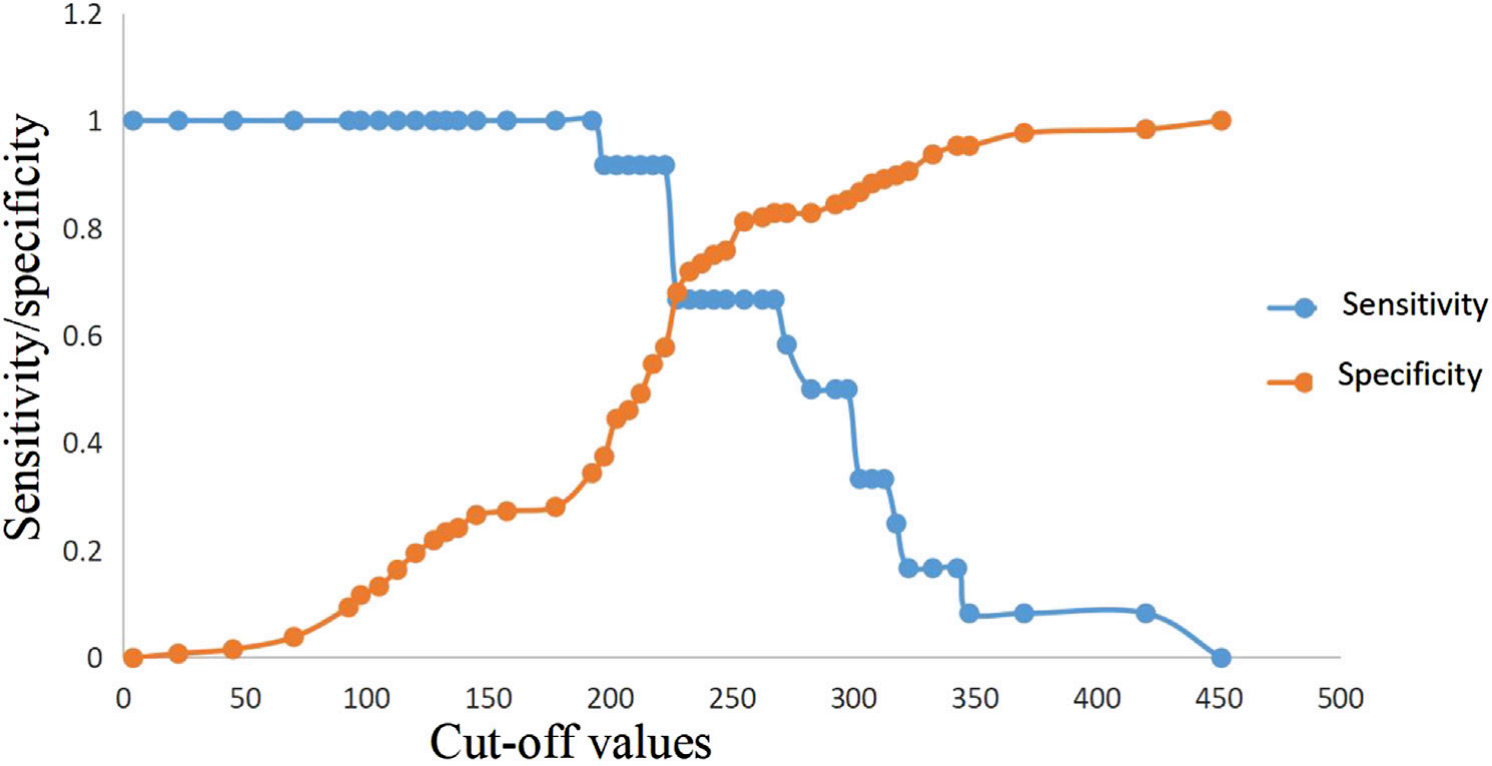
Sensitivity and specificity curves of PASS score in in-hospital mortality prediction in the presence of different cut-off values. PASS: pancreatitis activity scoring system.

ROC curve analysis showed that lactic acid (AUC = 0.729, 95% CI: 0.559–0.900), procalcitonin (AUC = 0.710, 95% CI: 0.594–0.826), urea nitrogen (AUC = 0.774, 95% CI: 0.653–0.895), PASS (AUC = 0.780, 95% CI: 0.669–0.891), and urea nitrogen in combination with PASS (AUC = 0.796, 95% CI: 0.697–0.894) are predictors of poor prognosis. The sensitivity of lactic acid, procalcitonin, urea nitrogen and PASS in mortality prediction were 0.667, 0.917, 0.917 and 0.667, respectively. Their specificity were 0.836, 0.586, 0.687 and 0.828, respectively. The best cut-off values were 2.4 mmol/L, 2.0 ng/ml, 10.1 mmol/L and 267.5 points, respectively (Table 4; Figures 6, 7).

### Evaluation of the ranson score’s predictive value

Due to some missing data, POF, poor prognosis, and in-hospital mortality were predicted based on Ranson scores of 82 AP patients in this study. The results showed that the predictive efficacy of Ranson score for POF was (AUC = 0.775, 95% CI: 0.674–0.877). Its sensitivity and specificity were 0.685 and 0.786, respectively. The best cut-off value was 2.5 points. The predictive efficacy of Ranson score for poor prognosis was (AUC = 0.663, 95% CI: 0.540–0.785). Its sensitivity and specificity were 0.733 and 0.596, respectively. The best cut-off value was 2.5 points. The predictive power of the Ranson score for in-hospital mortality was (AUC = 0.570, 95% CI: 0.540–0.785). Its sensitivity and specificity were 0.373 and 0.767, respectively. The best cut-off was 3.5 points (Table 7).

**TABLE 7:** ROC curve analysis of Ranson score in 82 AP patients.

Ranson score	AUC	Cut-off level	Sensitivity	Specificity	Youden’s index
POF	0.775	2.5	0.685	0.786	0.471
Poor prognosis	0.663	2.5	0.733	0.596	0.329
In-hospital mortality	0.570	3.5	0.400	0.722	0.122

## Discussion

In recent years, the incidence of AP has increased annually. The mortality of patients with MSAP and SAP has remained high. Therefore, early prediction and intervention of AP progression to severe illness have always been the focus of clinicians (Stigliano et al., 2017). Some hematological indexes, such as urea nitrogen, lactic acid, high density lipoprotein cholesterol, procalcitonin, interleukin-17, and C-reactive protein, have successively been found to have the good power to predict AP outcomes (Jansen et al., 2009; Peng et al., 2015; Komolafe et al., 2017; Lin et al., 2017; Gao et al., 2018; Zhou et al., 2018; Shen et al., 2021). In addition, the Ranson, APACHEⅡ and BISAP scores were also used to predict the p AP progression (Nakhoda et al., 2017; Hagjer and Kumar, 2018; Harshit Kumar and Singh Griwan, 2018; Arif et al., 2019; Wan et al., 2019). Our research found that PASS was an independent risk factor for POF and poor prognosis in patients with MSAP and SAP. PASS also represented a risk factor for in-hospital mortality. In the single-index analysis, PASS was superior to lactic acid, procalcitonin, urea nitrogen and Ranson score in predicting POF and hospital mortality. PASS was only inferior to urea nitrogen in predicting poor prognosis. There is nearly no difference in AUC between PASS and BUN. The only difference may be induced by the relatively small samples of patients. And large-samples including multi-center clinical studies are needed for further research. This finding indicates that PASS might be of great clinical significance for early prediction and intervention in patients with MSAP and SAP. Therefore, a specific PASS threshold could be employed to evaluate the prognosis of AP patients.

PASS was markedly superior to Ranson score in predicting the outcomes of POF, poor prognosis and in-hospital mortality. Buxbaum et al. also found that PASS was more effective in predicting MSAP and SAP in AP patients than Ranson score (Buxbaum et al., 2018). However, this difference might be due to the limited amount of Ranson data from the patients in this study. Therefore, the comparison of prediction ability between the two can be further studied and explored.

Because of the unpredictable nature AP pathological development, assessment of prognosis only by one hematological index or score is not reliable. Multiple indexes are often required to evaluate patients with MSAP and SAP. In our study, the predictive model constructed by binary logistic showed that the ability of urea nitrogen combined with PASS to predict POF (AUC = 0.849, 95% CI: 0.779–0.920), poor prognosis (AUC = 0.801, 95% CI: 0.726–0.876) and in-hospital death (AUC = 0.796, 95% CI: 0.697–0.894) was significantly better than that of each factor alone. Urea nitrogen combined with PASS might have some clinical utility in predicting AP prognosis.

Previous studies mainly focused on the relationships of PASS with other complications, such as hospitalization time, readmission rate, and infectious pancreatic necrosis (Buxbaum et al., 2018; Ke et al., 2018). The advantage of our research is that there were a large number of patients with MSAP and SAP. Furthermore, the age, gender and other confounding factors of the patients were adjusted by binary logistic regression to focus on the relationship between PASS and the overall prognosis of patients with severe AP. Our study also has some limitations. First, this single-center study only evaluated patients admitted to the ICU of Shandong Provincial Hospital. Second, this study only evaluated patients with MSAP and SAP. Therefore, whether these conclusions can be applied to MAP patients requires further exploration. Due to lack of raw data in this study, the comparison between PASS and APACHEⅡ scores was not carried out. Some studies suggest that PASS is significantly superior to APACHEⅡ score in predicting infectious pancreatic necrosis and new-onset organ failure (Ke et al., 2018; Thiruvengadam et al., 2021). However, further studies are needed to compare the predictive efficiency between the two scores on in-hospital death, progression to POF, and poor prognosis.

## Conclusion

In summary, the first measurement of PASS in MSAP and SAP patients admitted to the ICU may be an independent risk factor for POF and poor prognosis, and a risk factor for in-hospital mortality. The predictive power of urea nitrogen combined with PASS for the three outcomes was better than that of each factor alone. Therefore, PASS may be used as an important parameter for clinical evaluation of AP patients.

## Data Availability

The original contributions presented in the study are included in the article/supplementary material further inquiries can be directed to the corresponding author.
